# (6-Oxido-2-oxo-1,2-dihydropyrimidine-5-carboxylato-κ^2^ *O* ^5^,*O* ^6^)(4-oxido-2-oxo-1,2-dihydropyrimidin-3-ium-5-carboxyl­ato-κ^2^ *O* ^4^,*O* ^5^)bis(1,10-phenanthroline-κ^2^ *N*,*N*′)erbium(III) dihydrate

**DOI:** 10.1107/S1600536808001487

**Published:** 2008-01-25

**Authors:** Hui-Hui Xing, Zi-Lu Chen, Seik Weng Ng

**Affiliations:** a College of Chemistry and Chemical Engineering, Guangxi Normal University, Gulin 541004, People’s Republic of China; b Department of Chemistry, University of Malaya, 50603, Kuala Lumpur, Malaysia

## Abstract

The erbium(III) atom in the title compound, [Er(C_5_H_2_N_2_O_4_)(C_5_H_3_N_2_O_4_)(C_12_H_8_N_2_)_2_]·2H_2_O, is located on a twofold rotation axis and chelated by two 1,10-phenanthroline heterocycles as well as by a 2,4-dihydroxy­pyrimidine-5-car­box­yl­ate monoanion and a 2,4-dihydroxy­pyrimidine-5-car­box­yl­ate dianion in a square-anti­prismatic coordination geometry.

## Related literature

For the structure of 2,4-dihydroxy­pyridimine-5-carboxylic acid, see: Law *et al.* (2004 ▶). This erbium compound is isostructural with the europium, terbium and ytterbium analogs; see Sun & Jin (2004 ▶) for their detailed description.
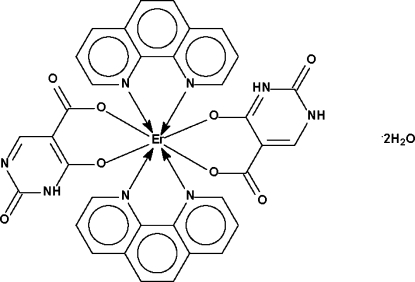


## Experimental

### 

#### Crystal data

[Er(C_5_H_2_N_2_O_4_)(C_5_H_3_N_2_O_4_)(C_12_H_8_N_2_)_2_]·2H_2_O
*M* *_r_* = 872.88
Monoclinic, 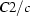

*a* = 17.1602 (7) Å
*b* = 14.4170 (6) Å
*c* = 13.2433 (5) Å
β = 101.159 (1)°
*V* = 3214.4 (2) Å^3^
*Z* = 4
Mo *K*α radiation
μ = 2.69 mm^−1^
*T* = 295 (2) K
0.18 × 0.10 × 0.08 mm

#### Data collection

Bruker APEXII diffractometer
Absorption correction: multi-scan (*SADABS*; Sheldrick, 1996 ▶) *T*_min_ = 0.645, *T*_max_ = 0.814
13567 measured reflections
3680 independent reflections
3495 reflections with *I* > 2σ(*I*)
*R*_int_ = 0.024

#### Refinement

*R*[*F* ^2^ > 2σ(*F* ^2^)] = 0.024
*wR*(*F* ^2^) = 0.061
*S* = 1.05
3680 reflections
240 parameters
H-atom parameters constrained
Δρ_max_ = 1.13 e Å^−3^
Δρ_min_ = −0.38 e Å^−3^

### 

Data collection: *APEX2* (Bruker, 2006 ▶); cell refinement: *SAINT* (Bruker, 2006 ▶); data reduction: *SAINT*; method used to solve structure: atomic coordinates taken from published analogs (Sun & Jin, 2004 ▶); program(s) used to refine structure: *SHELXL97* (Sheldrick, 2008 ▶); molecular graphics: *X-SEED* (Barbour, 2001 ▶); software used to prepare material for publication: *publCIF* (Westrip, 2008 ▶).

## Supplementary Material

Crystal structure: contains datablocks global, I. DOI: 10.1107/S1600536808001487/xu2400sup1.cif

Structure factors: contains datablocks I. DOI: 10.1107/S1600536808001487/xu2400Isup2.hkl

Additional supplementary materials:  crystallographic information; 3D view; checkCIF report

## Figures and Tables

**Table:** 

Er1—O2	2.297 (2)
Er1—O3	2.238 (2)
Er1—N3	2.558 (2)
Er1—N4	2.538 (2)

**Table:** 

O2—Er1—O2^i^	146.6 (1)
O2—Er1—O3	74.8 (1)
O2—Er1—O3^i^	81.6 (1)
O2—Er1—N3	74.5 (1)
O2—Er1—N3^i^	122.3 (1)
O2—Er1—N4	135.5 (1)
O2—Er1—N4^i^	74.6 (1)
O3—Er1—O3^i^	89.2 (1)
O3—Er1—N3	148.4 (1)
O3—Er1—N3^i^	79.0 (1)
O3—Er1—N4	147.2 (1)
O3—Er1—N4^i^	105.5 (1)
N3—Er1—N3^i^	124.7 (1)
N3—Er1—N4	64.4 (1)
N3—Er1—N4^i^	73.2 (1)
N4—Er1—N4^i^	77.9 (1)

Symmetry code: (i) 
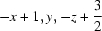
.

**Fig. 1.**
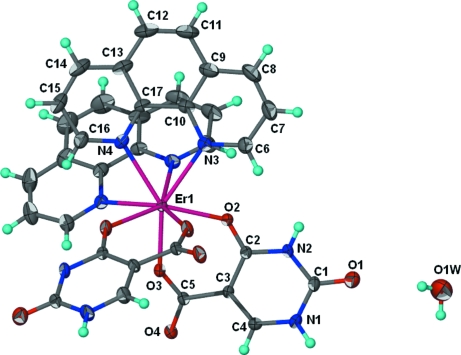
Thermal ellipsoid plot of Er(C12H8N2)2(C5H3N2O4)(C5H2N2O4).2H2O drawn at the 50% probability level. Hydrogen atoms are drawn as spheres of arbitrary radii.

**Fig. 2.**
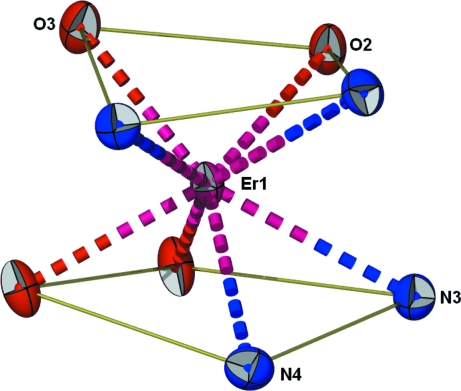
Square antiprismatic coordination geometry of Er.

## supplementary crystallographic information

### Comment

2,4-Dihydroxypyrimidine-5-carboxylic acid (uracil-5-carboxylic acid, isoorotic acid) in the form of the singly-, doubly- and triply-deprotonated ion furnishes a number of compounds with both main group and transition metals in which the anion functions in a variety of binding modes. The acid itself exists as hydrated molecules held together by extensive hydrogen bonds (Law *et al.*, 2004). The anion typically uses the 5-carboxylate and the 4-oxo/hydroxy oxygen atoms to chelate as this furnishes a six-membered chelate ring that confers stability.

The 1,10-phenanthroline-chelated rare-earth compounds, Ln(C_12_H_8_N_2_)_2_(C_5_H_3_N_2_O_4_)(C_5_H_2_N_2_O_4_)^.^2H_2_O (Ln = Eu, Tb and Yb) represent the first examples of mononuclear lanthanum derivatives of the acid (Sun & Jin, 2004). The present erbium analog is isostructural with these, whose structures have been described in detail.

### Experimental

2,4-Dihydroxypyrimidine-5-carboxylic acid (0.044 g, 0.25 mmol), erbium trichloride hexahydrate (0.096 g, 0.25 mmol), 1,10-phenanthroline (0.050 g, 0.25 mmol), sodium hydroxide (0.010 g, 0.25 mmol) and water (15 ml) was sealed in a 25-ml, Teflon-lined, stainless-steel Parr bomb. The bomb was heated to 383 K for 120 h. It was then cooled over 48 h to give red crystals in 90% yield. CH&N elemental analysis. Found/Calc. for C_34_H_25_ErN_8_O_10_: C 46.01; H 2.81, N 13.49% (46.78, 2.89, 12.84%).

### Refinement

Carbon-bound hydrogen atoms were generated geometrically, and were included in the refinements in the riding model approximation, as well the nitrogen-bound ones. The oxygen-bound ones were placed in chemically sensible positions on the basis of hydrogen bonding interactions. The final difference Fourier map had a large peak near Er1.

### Crystal data

**Table:**

[Er(C_5_H_2_N_2_O_4_)(C_5_H_3_N_2_O_4_)(C_12_H_8_N_2_)_2_]·2H_2_O	*F*(000) = 1732
*M**_r_* = 872.88	*D*_x_ = 1.804 Mg m^−^^3^
Monoclinic, *C*2/*c*	Mo *K*α radiation, λ = 0.71073 Å
Hall symbol: -C 2yc	Cell parameters from 8216 reflections
*a* = 17.1602 (7) Å	θ = 2.3–28.5°
*b* = 14.4170 (6) Å	µ = 2.69 mm^−^^1^
*c* = 13.2433 (5) Å	*T* = 295 K
β = 101.159 (1)°	Prism, red
*V* = 3214.4 (2) Å^3^	0.18 × 0.10 × 0.08 mm
*Z* = 4	

### Data collection

**Table:**

Bruker APEXII diffractometer	3680 independent reflections
Radiation source: fine-focus sealed tube	3495 reflections with *I* > 2σ(*I*)
graphite	*R*_int_ = 0.024
φ and ω scans	θ_max_ = 27.5°, θ_min_ = 1.9°
Absorption correction: multi-scan (*SADABS*; Sheldrick, 1996)	*h* = −22→22
*T*_min_ = 0.645, *T*_max_ = 0.814	*k* = −18→18
13567 measured reflections	*l* = −17→17

### Refinement

**Table:**

Refinement on *F*^2^	Primary atom site location: structure-invariant direct methods
Least-squares matrix: full	Secondary atom site location: difference Fourier map
*R*[*F*^2^ > 2σ(*F*^2^)] = 0.024	Hydrogen site location: inferred from neighbouring sites
*wR*(*F*^2^) = 0.061	H-atom parameters constrained
*S* = 1.05	*w* = 1/[σ^2^(*F*_o_^2^) + (0.0387*P*)^2^ + 2.1981*P*] where *P* = (*F*_o_^2^ + 2*F*_c_^2^)/3
3680 reflections	(Δ/σ)_max_ = 0.001
240 parameters	Δρ_max_ = 1.13 e Å^−^^3^
0 restraints	Δρ_min_ = −0.38 e Å^−^^3^

### Fractional atomic coordinates and isotropic or equivalent isotropic displacement parameters (Å^2^)

**Table:**

	*x*	*y*	*z*	*U*_iso_*/*U*_eq_	Occ. (<1)
Er1	0.5000	0.612675 (9)	0.7500	0.02099 (6)	
O1	0.26939 (16)	0.37986 (14)	0.36337 (19)	0.0436 (6)	
O2	0.40471 (11)	0.56690 (12)	0.61245 (13)	0.0293 (4)	
O3	0.43054 (11)	0.50209 (13)	0.81183 (13)	0.0312 (4)	
O4	0.34769 (14)	0.39182 (12)	0.84072 (15)	0.0341 (5)	
O1W	0.1905 (2)	0.3113 (2)	0.1608 (2)	0.0912 (11)	
H1W1	0.2254	0.3253	0.2146	0.137*	
H1W2	0.2024	0.2603	0.1354	0.137*	
N1	0.28276 (16)	0.33178 (16)	0.53134 (18)	0.0367 (5)	
H1N	0.2588	0.2802	0.5137	0.055*	0.50
N2	0.33356 (13)	0.47408 (15)	0.49382 (16)	0.0276 (5)	
H2N	0.3385	0.5157	0.4489	0.041*	
N3	0.53478 (13)	0.69505 (15)	0.59349 (17)	0.0278 (5)	
N4	0.59394 (13)	0.74961 (15)	0.78945 (17)	0.0289 (5)	
C1	0.29379 (18)	0.39350 (17)	0.4575 (2)	0.0295 (6)	
C2	0.36564 (14)	0.49369 (17)	0.59430 (18)	0.0229 (5)	
C3	0.35025 (15)	0.42826 (17)	0.66835 (18)	0.0248 (5)	
C4	0.30868 (18)	0.3506 (2)	0.6303 (2)	0.0342 (6)	
H4	0.2977	0.3074	0.6777	0.041*	
C5	0.37729 (15)	0.44133 (17)	0.78069 (18)	0.0247 (5)	
C6	0.50987 (17)	0.6671 (2)	0.4970 (2)	0.0346 (6)	
H6	0.4906	0.6070	0.4856	0.042*	
C7	0.5112 (2)	0.7236 (3)	0.4122 (2)	0.0486 (8)	
H7	0.4946	0.7008	0.3459	0.058*	
C8	0.5368 (2)	0.8118 (3)	0.4271 (3)	0.0556 (9)	
H8	0.5352	0.8511	0.3710	0.067*	
C9	0.5659 (2)	0.8442 (2)	0.5267 (3)	0.0438 (7)	
C10	0.56432 (16)	0.78248 (18)	0.6084 (2)	0.0307 (6)	
C11	0.5963 (2)	0.9365 (3)	0.5497 (3)	0.0598 (10)	
H11	0.5950	0.9787	0.4963	0.072*	
C12	0.6260 (2)	0.9627 (2)	0.6459 (3)	0.0586 (10)	
H12	0.6457	1.0226	0.6582	0.070*	
C13	0.6285 (2)	0.9006 (2)	0.7304 (3)	0.0414 (7)	
C14	0.6628 (2)	0.9230 (2)	0.8322 (3)	0.0501 (9)	
H14	0.6846	0.9816	0.8475	0.060*	
C15	0.6646 (2)	0.8603 (2)	0.9083 (3)	0.0464 (8)	
H15	0.6891	0.8743	0.9755	0.056*	
C16	0.62887 (17)	0.7737 (2)	0.8840 (2)	0.0365 (6)	
H16	0.6297	0.7310	0.9369	0.044*	
C17	0.59569 (16)	0.81116 (18)	0.7125 (2)	0.0289 (5)	

### Atomic displacement parameters (Å^2^)

**Table:**

	*U*^11^	*U*^22^	*U*^33^	*U*^12^	*U*^13^	*U*^23^
Er1	0.02696 (10)	0.01677 (9)	0.01709 (9)	0.000	−0.00108 (6)	0.000
O1	0.0548 (14)	0.0334 (12)	0.0366 (12)	−0.0041 (9)	−0.0064 (11)	0.0014 (8)
O2	0.0391 (10)	0.0236 (9)	0.0213 (9)	−0.0088 (8)	−0.0037 (7)	0.0029 (7)
O3	0.0431 (11)	0.0295 (10)	0.0185 (8)	−0.0109 (8)	0.0001 (8)	−0.0010 (7)
O4	0.0482 (13)	0.0337 (11)	0.0205 (10)	−0.0117 (8)	0.0067 (9)	0.0007 (7)
O1W	0.138 (3)	0.0561 (18)	0.0636 (19)	0.0005 (19)	−0.020 (2)	0.0078 (15)
N1	0.0543 (15)	0.0264 (11)	0.0258 (11)	−0.0119 (11)	−0.0012 (10)	−0.0033 (9)
N2	0.0369 (12)	0.0260 (11)	0.0170 (10)	−0.0052 (9)	−0.0019 (8)	0.0019 (8)
N3	0.0300 (11)	0.0262 (11)	0.0268 (11)	0.0003 (9)	0.0044 (9)	0.0020 (9)
N4	0.0279 (11)	0.0259 (11)	0.0318 (12)	−0.0052 (9)	0.0032 (9)	−0.0043 (9)
C1	0.0366 (15)	0.0267 (13)	0.0216 (13)	0.0007 (10)	−0.0037 (11)	−0.0030 (9)
C2	0.0263 (12)	0.0213 (11)	0.0193 (11)	0.0005 (9)	−0.0001 (9)	0.0004 (9)
C3	0.0339 (13)	0.0218 (12)	0.0179 (11)	−0.0048 (10)	0.0030 (10)	−0.0021 (9)
C4	0.0488 (17)	0.0265 (14)	0.0258 (13)	−0.0117 (12)	0.0038 (12)	0.0025 (11)
C5	0.0325 (13)	0.0224 (12)	0.0185 (11)	−0.0001 (10)	0.0033 (9)	0.0000 (9)
C6	0.0353 (14)	0.0391 (15)	0.0288 (14)	−0.0033 (12)	0.0046 (11)	−0.0005 (12)
C7	0.0508 (19)	0.069 (2)	0.0261 (15)	−0.0105 (17)	0.0074 (13)	0.0035 (15)
C8	0.064 (2)	0.065 (2)	0.0360 (18)	−0.0098 (19)	0.0076 (16)	0.0225 (16)
C9	0.0465 (18)	0.0383 (17)	0.0466 (18)	−0.0032 (14)	0.0093 (14)	0.0143 (14)
C10	0.0317 (13)	0.0262 (13)	0.0349 (14)	−0.0009 (11)	0.0085 (11)	0.0053 (11)
C11	0.073 (3)	0.040 (2)	0.066 (3)	−0.0157 (18)	0.014 (2)	0.0201 (18)
C12	0.066 (2)	0.0325 (18)	0.079 (3)	−0.0160 (16)	0.017 (2)	0.0063 (17)
C13	0.0412 (17)	0.0255 (14)	0.060 (2)	−0.0080 (12)	0.0156 (15)	−0.0060 (13)
C14	0.0486 (19)	0.0355 (17)	0.068 (2)	−0.0169 (15)	0.0155 (17)	−0.0174 (17)
C15	0.0415 (17)	0.0477 (18)	0.049 (2)	−0.0117 (15)	0.0063 (15)	−0.0215 (16)
C16	0.0352 (15)	0.0401 (16)	0.0330 (15)	−0.0091 (12)	0.0036 (12)	−0.0077 (12)
C17	0.0269 (13)	0.0220 (12)	0.0383 (15)	−0.0022 (10)	0.0075 (11)	−0.0009 (10)

### Geometric parameters (Å, °)

**Table:**

Er1—O2	2.297 (2)	C2—C3	1.422 (3)
Er1—O2^i^	2.297 (2)	C3—C4	1.371 (4)
Er1—O3	2.238 (2)	C3—C5	1.482 (3)
Er1—O3^i^	2.238 (2)	C4—H4	0.9300
Er1—N3	2.558 (2)	C6—C7	1.391 (4)
Er1—N3^i^	2.558 (2)	C6—H6	0.9300
Er1—N4	2.538 (2)	C7—C8	1.346 (5)
Er1—N4^i^	2.538 (2)	C7—H7	0.9300
O1—C1	1.252 (4)	C8—C9	1.398 (5)
O2—C2	1.248 (3)	C8—H8	0.9300
O3—C5	1.275 (3)	C9—C10	1.405 (4)
O4—C5	1.247 (3)	C9—C11	1.440 (5)
O1W—H1W1	0.85	C10—C17	1.441 (4)
O1W—H1W2	0.85	C11—C12	1.332 (6)
N1—C4	1.327 (3)	C11—H11	0.9300
N1—C1	1.362 (4)	C12—C13	1.428 (5)
N1—H1N	0.8600	C12—H12	0.9300
N2—C2	1.368 (3)	C13—C14	1.400 (5)
N2—C1	1.385 (3)	C13—C17	1.409 (4)
N2—H2N	0.8600	C14—C15	1.349 (6)
N3—C6	1.329 (3)	C14—H14	0.9300
N3—C10	1.359 (3)	C15—C16	1.401 (4)
N4—C16	1.326 (4)	C15—H15	0.9300
N4—C17	1.356 (3)	C16—H16	0.9300
			
O2—Er1—O2^i^	146.6 (1)	N1—C4—H4	117.2
O2—Er1—O3	74.8 (1)	C3—C4—H4	117.2
O2—Er1—O3^i^	81.6 (1)	O4—C5—O3	122.8 (2)
O2—Er1—N3	74.5 (1)	O4—C5—C3	118.7 (2)
O2—Er1—N3^i^	122.3 (1)	O3—C5—C3	118.5 (2)
O2—Er1—N4	135.5 (1)	N3—C6—C7	123.1 (3)
O2—Er1—N4^i^	74.6 (1)	N3—C6—H6	118.5
O3—Er1—O3^i^	89.2 (1)	C7—C6—H6	118.5
O3—Er1—N3	148.4 (1)	C8—C7—C6	119.4 (3)
O3—Er1—N3^i^	79.0 (1)	C8—C7—H7	120.3
O3—Er1—N4	147.2 (1)	C6—C7—H7	120.3
O3—Er1—N4^i^	105.5 (1)	C7—C8—C9	120.0 (3)
N3—Er1—N3^i^	124.7 (1)	C7—C8—H8	120.0
N3—Er1—N4	64.4 (1)	C9—C8—H8	120.0
N3—Er1—N4^i^	73.2 (1)	C8—C9—C10	117.3 (3)
N4—Er1—N4^i^	77.9 (1)	C8—C9—C11	123.9 (3)
C2—O2—Er1	131.99 (15)	C10—C9—C11	118.9 (3)
C5—O3—Er1	140.34 (16)	N3—C10—C9	122.6 (3)
H1W1—O1W—H1W2	110.3	N3—C10—C17	117.7 (2)
C4—N1—C1	120.6 (2)	C9—C10—C17	119.7 (3)
C4—N1—H1N	119.7	C12—C11—C9	121.4 (3)
C1—N1—H1N	119.7	C12—C11—H11	119.3
C2—N2—C1	126.1 (2)	C9—C11—H11	119.3
C2—N2—H2N	116.9	C11—C12—C13	121.3 (3)
C1—N2—H2N	116.9	C11—C12—H12	119.3
C6—N3—C10	117.5 (2)	C13—C12—H12	119.3
C6—N3—Er1	123.81 (18)	C14—C13—C17	116.9 (3)
C10—N3—Er1	116.89 (17)	C14—C13—C12	123.6 (3)
C16—N4—C17	117.8 (2)	C17—C13—C12	119.5 (3)
C16—N4—Er1	123.46 (19)	C15—C14—C13	120.6 (3)
C17—N4—Er1	117.38 (16)	C15—C14—H14	119.7
O1—C1—N1	123.1 (2)	C13—C14—H14	119.7
O1—C1—N2	121.6 (2)	C14—C15—C16	118.7 (3)
N1—C1—N2	115.2 (2)	C14—C15—H15	120.7
O2—C2—N2	117.5 (2)	C16—C15—H15	120.7
O2—C2—C3	126.3 (2)	N4—C16—C15	123.2 (3)
N2—C2—C3	116.1 (2)	N4—C16—H16	118.4
C4—C3—C2	116.2 (2)	C15—C16—H16	118.4
C4—C3—C5	120.8 (2)	N4—C17—C13	122.6 (3)
C2—C3—C5	123.0 (2)	N4—C17—C10	118.2 (2)
N1—C4—C3	125.5 (3)	C13—C17—C10	119.2 (3)
			
O3—Er1—O2—C2	−23.0 (2)	O2—C2—C3—C4	−177.3 (3)
O3^i^—Er1—O2—C2	68.4 (2)	N2—C2—C3—C4	3.4 (4)
O2^i^—Er1—O2—C2	23.4 (2)	O2—C2—C3—C5	2.4 (4)
N4—Er1—O2—C2	172.0 (2)	N2—C2—C3—C5	−176.9 (2)
N4^i^—Er1—O2—C2	−134.2 (2)	C1—N1—C4—C3	−2.7 (5)
N3^i^—Er1—O2—C2	−89.1 (2)	C2—C3—C4—N1	0.6 (5)
N3—Er1—O2—C2	149.3 (2)	C5—C3—C4—N1	−179.1 (3)
O3^i^—Er1—O3—C5	−72.0 (3)	Er1—O3—C5—O4	−175.83 (19)
O2—Er1—O3—C5	9.5 (3)	Er1—O3—C5—C3	5.0 (4)
O2^i^—Er1—O3—C5	−146.7 (3)	C4—C3—C5—O4	−15.3 (4)
N4—Er1—O3—C5	169.9 (2)	C2—C3—C5—O4	165.0 (3)
N4^i^—Er1—O3—C5	78.3 (3)	C4—C3—C5—O3	163.9 (3)
N3^i^—Er1—O3—C5	137.6 (3)	C2—C3—C5—O3	−15.7 (4)
N3—Er1—O3—C5	−4.7 (3)	C10—N3—C6—C7	−1.4 (4)
O3—Er1—N3—C6	−6.7 (3)	Er1—N3—C6—C7	162.9 (2)
O3^i^—Er1—N3—C6	63.3 (2)	N3—C6—C7—C8	−1.8 (5)
O2—Er1—N3—C6	−20.9 (2)	C6—C7—C8—C9	3.6 (6)
O2^i^—Er1—N3—C6	127.3 (2)	C7—C8—C9—C10	−2.2 (5)
N4—Er1—N3—C6	176.5 (2)	C7—C8—C9—C11	178.5 (4)
N4^i^—Er1—N3—C6	−99.1 (2)	C6—N3—C10—C9	2.9 (4)
N3^i^—Er1—N3—C6	−139.7 (2)	Er1—N3—C10—C9	−162.5 (2)
O3—Er1—N3—C10	157.69 (17)	C6—N3—C10—C17	−176.4 (2)
O3^i^—Er1—N3—C10	−132.33 (19)	Er1—N3—C10—C17	18.2 (3)
O2—Er1—N3—C10	143.5 (2)	C8—C9—C10—N3	−1.1 (5)
O2^i^—Er1—N3—C10	−68.3 (2)	C11—C9—C10—N3	178.3 (3)
N4—Er1—N3—C10	−19.07 (17)	C8—C9—C10—C17	178.2 (3)
N4^i^—Er1—N3—C10	65.31 (19)	C11—C9—C10—C17	−2.4 (5)
N3^i^—Er1—N3—C10	24.68 (17)	C8—C9—C11—C12	−177.4 (4)
O3—Er1—N4—C16	8.6 (3)	C10—C9—C11—C12	3.2 (6)
O3^i^—Er1—N4—C16	−105.1 (2)	C9—C11—C12—C13	−0.6 (7)
O2—Er1—N4—C16	161.2 (2)	C11—C12—C13—C14	176.5 (4)
O2^i^—Er1—N4—C16	−36.1 (2)	C11—C12—C13—C17	−2.7 (6)
N4^i^—Er1—N4—C16	108.5 (2)	C17—C13—C14—C15	0.7 (5)
N3^i^—Er1—N4—C16	41.9 (2)	C12—C13—C14—C15	−178.6 (4)
N3—Er1—N4—C16	−174.5 (2)	C13—C14—C15—C16	−2.3 (5)
O3—Er1—N4—C17	−157.77 (17)	C17—N4—C16—C15	2.3 (4)
O3^i^—Er1—N4—C17	88.48 (19)	Er1—N4—C16—C15	−164.0 (2)
O2—Er1—N4—C17	−5.2 (2)	C14—C15—C16—N4	0.8 (5)
O2^i^—Er1—N4—C17	157.4 (2)	C16—N4—C17—C13	−4.1 (4)
N4^i^—Er1—N4—C17	−57.88 (17)	Er1—N4—C17—C13	163.1 (2)
N3^i^—Er1—N4—C17	−124.5 (2)	C16—N4—C17—C10	174.5 (2)
N3—Er1—N4—C17	19.09 (18)	Er1—N4—C17—C10	−18.3 (3)
C4—N1—C1—O1	−178.8 (3)	C14—C13—C17—N4	2.6 (4)
C4—N1—C1—N2	0.5 (4)	C12—C13—C17—N4	−178.1 (3)
C2—N2—C1—O1	−176.8 (3)	C14—C13—C17—C10	−175.9 (3)
C2—N2—C1—N1	3.9 (4)	C12—C13—C17—C10	3.4 (4)
Er1—O2—C2—N2	−158.37 (17)	N3—C10—C17—N4	−0.1 (4)
Er1—O2—C2—C3	22.3 (4)	C9—C10—C17—N4	−179.4 (3)
C1—N2—C2—O2	174.7 (3)	N3—C10—C17—C13	178.5 (3)
C1—N2—C2—C3	−5.9 (4)	C9—C10—C17—C13	−0.8 (4)

Symmetry codes: (i) −*x*+1, *y*, −*z*+3/2.

### Hydrogen-bond geometry (Å, °)

**Table:**

*D*—H···*A*	*D*—H	H···*A*	*D*···*A*	*D*—H···*A*
N1—H1n···N1^ii^	0.86	1.82	2.675 (5)	174
N2—H2n···O4^iii^	0.86	1.99	2.846 (3)	178
O1w—H1w1···O1	0.85	2.12	2.933 (4)	157
O1w—H1w2···O4^ii^	0.85	2.40	3.000 (4)	128

Symmetry codes: (ii) −*x*+1/2, −*y*+1/2, −*z*+1; (iii) *x*, −*y*+1, *z*−1/2.
